# Single-step method for β-galactosidase assays in *Escherichia coli* using a 96-well microplate reader

**DOI:** 10.1016/j.ab.2016.03.017

**Published:** 2016-06-15

**Authors:** Jorrit Schaefer, Goran Jovanovic, Ioly Kotta-Loizou, Martin Buck

**Affiliations:** Department of Life Sciences, Faculty of Natural Sciences, Imperial College London, London SW7 2AZ, UK

**Keywords:** LacZ, B-Galactosidase (Bgal), β-Galactosidase, Microplate reader, ONPG, *o*-nitrophenyl-ß-d-galactopyranoside, SDS, sodium dodecyl sulfate, Bgal, B-galactosidase

## Abstract

Historically, the *lacZ* gene is one of the most universally used reporters of gene expression in molecular biology. Its activity can be quantified using an artificial substrate, *o*-nitrophenyl-ß-d-galactopyranoside (ONPG). However, the traditional method for measuring LacZ activity (first described by J. H. Miller in 1972) can be challenging for a large number of samples, is prone to variability, and involves hazardous compounds for lysis (e.g., chloroform, toluene).

Here we describe a single-step assay using a 96-well microplate reader with a proven alternative cell permeabilization method. This modified protocol reduces handling time by 90%.

Various β-galactosidase protocols for bacteria have been described, adapting some of the Miller method [1] steps for use in plate readers [2], [3], [4], [5]. However, these methods include many of the drawbacks inherent to the original method and remain labor-intensive.

One of the challenges in further speeding up this assay is the cell permeabilization stage, which is required for the *o*-nitrophenyl-ß-d-galactopyranoside (ONPG) substrate to enter the cell and interact with β-galactosidase. This typically requires the transfer of cultures due to the fact that permeabilization is normally performed using chloroform/sodium dodecyl sulfate (SDS) or toluene [1], [6], which can interfere with the optical density readings in standard microtiter plates. Deep well nonreactive polypropylene blocks have been suggested [5]; however, the organic solvents were reported to be difficult to manipulate using multichannel pipettes [7].

An alternative permeabilization method was proposed using PopCulture reagent [7], a compound used in protein purification. PopCulture reagent punctures the cell wall without denaturing soluble proteins or interfering with optical density readings, with the β-galactosidase remaining stable for up to 18 h [7]. The cell lysis efficiency can be further enhanced by the addition of chicken egg white lysozyme, which hydrolyzes the peptidoglycan in cell walls [8]. This protocol was shown to produce similar results to the traditional chloroform/SDS method used for cell lysis [7]. This approach allowed for kinetic readings rather than endpoint readings, obviating the need for stopping the reaction with Na_2_CO_3_ and thereby improving accuracy.

Although this new permeabilization method has improved accuracy of the assay and reduced handling time for a large number of samples, the time taken to process smaller numbers of samples remains largely unchanged. Here we describe a streamlined version of these methods to condense the assay from several liquid handling steps into a single-step assay, decreasing the labor intensity irrespective of sample size.

The one-step approach aims to combine (i) OD_600_ measurement, (ii) cell permeabilization, (iii) ONPG breakdown, and (iv) kinetic OD_420_ quantification into a single step. The approach involves transferring 80 μl of cells and 120 μl of custom B-galactosidase (Bgal) mix (60 mM Na_2_HPO_4_, 40 mM NaH_2_PO_4_, 10 mM KCl, 1 mM MgSO_4_, 36 mM β-mercaptoethanol, 166 μl/ml T7 lysozyme, 1.1 mg/ml ONPG, and 6.7% PopCulture reagent) to a microtiter plate, followed by kinetic OD_420_ and OD_600_ quantification on a FLUOstar Omega Microplate Reader (BMG Labtech). These are then converted into Miller units using MARS Data Analysis software. A more detailed protocol to run this assay (including FLUOstar Omega Microplate Reader script) is also available (see Ref. [9], Supplementary Data A and B).

To combine several liquid handling steps into a single one, cell permeabilization must be rapid. Slow permeabilization could otherwise reduce ONPG availability within the cell and have adverse effects on OD_420_ production and Miller units. We showed that permeabilization is immediate using the one-step β-galactosidase method, with a linear OD_420_ increase over time being observed throughout the assay until ONPG is depleted (Fig. 1A). Therefore, any lack of cell permeabilization does not impact ONPG availability significantly given that no initial lag phase is observed for OD_420_ readings (Fig. 1A). Moreover, similar Miller unit values were obtained using the diluted cultures (Fig. 1B), suggesting that the results from this modified assay are consistent and scalable.

Second, the one-step assay also determines OD_600_ readings at the start of the assay to avoid additional liquid handling steps. We demonstrated that cell cultures diluted in rich medium (LB medium) versus the Bgal mix are comparable over an OD_600_ range of 0.1–1.2 (Fig. 1C). This covers the range of cell densities used in a typical β-galactosidase assay, indicating that the OD_600_ measurement of cells diluted Bgal mix could potentially be used as a substitute for its quantification in LB medium.

Finally, data comparisons between the traditional fully manual assay and the one-step method were not significantly different over a range of activities (Fig. 1D; see Ref. [9], Table 1). Moderate differences observed between the two methods can likely be attributed to minor discrepancies in OD_600_ measurements and the lack of Na_2_CO_3_ (stop solution), which increases the OD_420_ slightly.

Overall, the single-step β-galactosidase assay yields both consistent and accurate results over the range of cell densities and LacZ levels typically tested with a standard β-galactosidase assay and is a suitable faster and safer alternative to the traditional method (or current methods used).

## Figures and Tables

**Fig.1:**
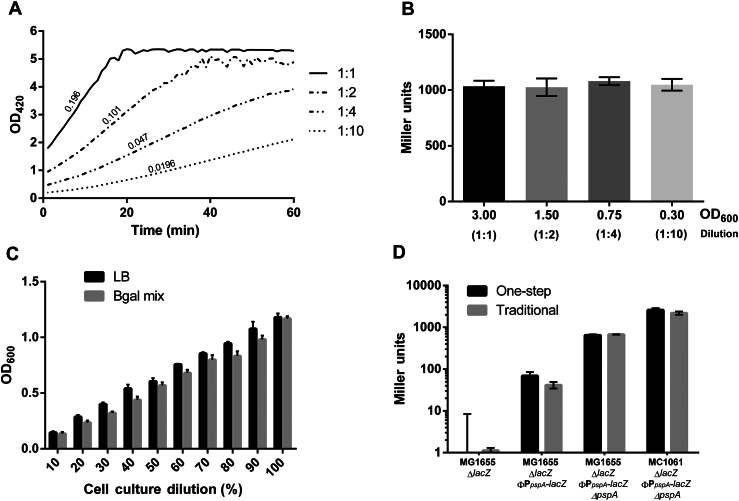
OD_420_ and OD_600_ measurements are linear over the ranges used in the one-step assay. (A) OD_420_ readings with the one-step assay were linear over time until ONPG was limiting, an OD_420_ of approximately 5. OD_420_/min values for undiluted cultures (1:1) and diluted cultures (1:2, 1:4, and 1:10) were directly proportional. (B) Each of the dilutions was assayed, and Miller unit activities were found to be highly similar over an OD_600_ range of 0.3–3.0. (C) OD_600_ values measured in LB medium or B-gal mix were both comparable and linear on dilution over an OD_600_ range of 0.12–1.2. (D) Miller unit values were not statistically different (Student's *t*-test, *P* > 0.05) in the traditional and one-step assays for four different strains, with varying levels of β-galactosidase activity. Error bars represent standard deviations of experiments performed in triplicate.
